# Different Effects of Induced Plant Defense on Population Densities of Specialist and Generalist Insects: An Explanation by Mathematical Modeling

**DOI:** 10.1002/ece3.74235

**Published:** 2026-09-09

**Authors:** Suman Chakraborty, Stefan Schuster

**Affiliations:** ^1^ Department of Bioinformatics Matthias Schleiden Institute, Friedrich Schiller University Jena Jena Germany; ^2^ International Max Planck Research School ‘Chemical Communication in Ecological Systems’ Jena Germany; ^3^ Department of Plant Protection Biology Swedish University of Agricultural Sciences Alnarp Sweden

**Keywords:** generalist herbivore, global stability, induced plant defense, natural enemies, nonautonomous ordinary differential equations, specialist herbivore, tritrophic interaction

## Abstract

In the wild, plant‐insect interactions are highly influenced by plant defense chemicals. These chemicals (or toxins) are usually harmful to attacking insect herbivores, especially generalists. Plant defense is categorized into two types: Constitutive and induced defense. Constitutive defense is present in plants from germination on, while induced defense is activated by biotic stress, such as herbivory, or abiotic stress. This work focuses on modeling the multifaceted ecological roles of defense chemicals and investigates how the community structures of specialists and generalists in an ecosystem are determined by induced defense. The ecological roles of defense are very diverse. Specialist insects are well adapted to plant toxins and thus become less affected than generalists. Specialists are even attracted by the defense chemicals, although both insect groups could be killed at high concentrations. Moreover, natural enemies of insects are recruited by plants through toxins. In this study, these fundamental ecological functions of plant defense are expressed by a deterministic model based on nonautonomous ordinary differential equations. By analyzing this model, we obtain a stable non‐zero equilibrium for specialists and a zero equilibrium for generalists at high concentrations of induced defense. Thus, it is proved theoretically that specialists are endemic and generalists are non‐endemic in the wild, a common real‐world situation, due to the defense induction in plants.

## Graphical abstract

Induced defense chemicals in plants determine the community structure of different insect (herbivore) groups. In particular, specialists are endemic and generalists are non‐endemic in the wild because of the ecological roles of induced secondary metabolites.

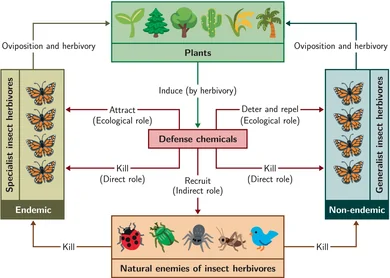

## 1 Introduction

Plants produce secondary metabolites that do not usually aid them in photosynthesis, primary growth, development, reproduction, etc. (Alvarez 2014; Teoh 2016). Nevertheless, these chemicals perform important functions in plant‐environment interactions, acting, for example, as odorants and pigments for attracting pollinators, etc. (War et al. 2019; Shivanna 2025). A multitude of secondary metabolites repel or poison insect herbivores in the wild and are thus classified as defense chemicals (Halkier and Gershenzon 2006; Wittstock and Gershenzon 2002; Wittstock et al. 2003; Wittstock and Burow 2010; Vickery 2010; Chakraborty et al. 2023a, 2024, 2023b; Jeschke et al. 2016, 2017, 2021; Zalucki et al. 2021; Chakraborty and Schuster 2024). Examples include alkaloids, such as cocaine, caffeine, nicotine, morphine, and atropine (Wink 2010; Wei et al. 2019); glucosinolates (Textor and Gershenzon 2009); terpenoids (Ninkuu et al. 2021) and many non‐proteinogenic amino acids (Fichtner et al. 2017).

Plants store defense chemicals constitutively; that is, a certain amount of defense is present in plants during the normal course of development after germination (Chakraborty et al. 2023a, 2024; Van Der Meijden 1996; Hopkins et al. 2009; Dicke and Baldwin 2010; Moreira et al. 2018). Constitutive defense provides fundamental protection to plants, which can prevent insects from selecting plants for oviposition (Chakraborty et al. 2023a, 2024; Van Der Meijden 1996). Under biotic (or abiotic) stress, plants increase these defense compounds to a higher level, called induced defense (Textor and Gershenzon 2009; Dicke and Baldwin 2010; Moreira et al. 2018; Dicke 1998; Sharma et al. 2022). Herbivory‐induced defense in plants against biotic stress is widely studied (Textor and Gershenzon 2009; Dicke and Baldwin 2010; Dicke 1998; Ali and Agrawal 2012; Halitschke et al. 2001). For example, herbivory by generalist insects induces nicotine in tobacco plants (Halitschke et al. 2001). The larvae of *Plutella xylostella* (diamondback moth) and *Pieris rapae* (small white) induce glucosinolates (by herbivory) in plants of the Brassicaceae family (Textor and Gershenzon 2009). Further examples of glucosinolates induction are documented in the literature (Textor and Gershenzon 2009; Ali and Agrawal 2012).

Generally, (extreme) specialists feed on one or a limited number of species of host plants, while (extreme) generalists feed on plants from many different families. However, these categories could be relative. A given herbivore can be described as a “generalist” if it feeds on a wider range of host plant species compared to another species; this same species may be regarded to be more specialized if it has a smaller set of host plants compared to a third one. Nevertheless, for simplicity's sake and in accordance with earlier approaches (Chakraborty et al. 2023a; Van Der Meijden 1996; Lankau 2007), insect herbivores are here categorized into two distinct groups: Specialists and generalists.

Specialist insects are well adapted to specific secondary metabolites (Ali and Agrawal 2012; Sun et al. 2019; Rohr et al. 2011; Sarosh et al. 2010). In addition, specialists use these defense compounds as a cue for oviposition and feeding (Bidart‐Bouzat and Kliebenstein 2008; Badenes‐Pérez et al. 2020; Macel 2011). For example, glucosinolates stimulate oviposition by specialist insects, such as *Pieris rapae, Plutella xylostella*, and *Pieris brassicaceae* in Brassicaceae (Bidart‐Bouzat and Kliebenstein 2008; Badenes‐Pérez et al. 2020; Raybould and Moyes 2001; Fei et al. 2017). Indioside D stimulates oviposition by *Manduca sexta* (tobacco hawk moth) in solanaceous plants (such as tomato and tobacco) (Del Campo et al. 2001). Generally, specialists are endemic on the plants of their preferred secondary metabolites, where endemic here means that specialists tend to remain on their preferred host plants. The term “endemic” is not primarily meant here in the geographic sense. Such plants are called primary hosts because specialists regularly oviposit on these plants (Devictor et al. 2010; Büchi and Vuilleumier 2014; Kneitel 2018). In contrast, generalist insects do not have primary hosts. Instead, they rely on temporary host plants of various families, meaning that insects do not oviposit and feed on them regularly (Walter and Benfield 1994; Wang et al. 2017; Rafter and Walter 2020). Such selection behavior makes generalists non‐endemic in the wild (Walter and Benfield 1994; Wang et al. 2017; Rafter and Walter 2020) because they forage across various plant families via host switching (Chakraborty et al. 2024; Devictor et al. 2010; Büchi and Vuilleumier 2014; Kneitel 2018). That occurs because generalists are not well adapted (compared to specialists) to plant toxins (Jeschke et al. 2017, 2021; Zalucki et al. 2021; Schramm et al. 2012) and are deterred by defense compounds (Chakraborty et al. 2023a, 2024; Van Der Meijden 1996). Moreover, their selection behavior is strongly influenced by nutritional factors and host availability (Walter and Benfield 1994; Wang et al. 2017; Rafter and Walter 2020).

**FIGURE 1:**
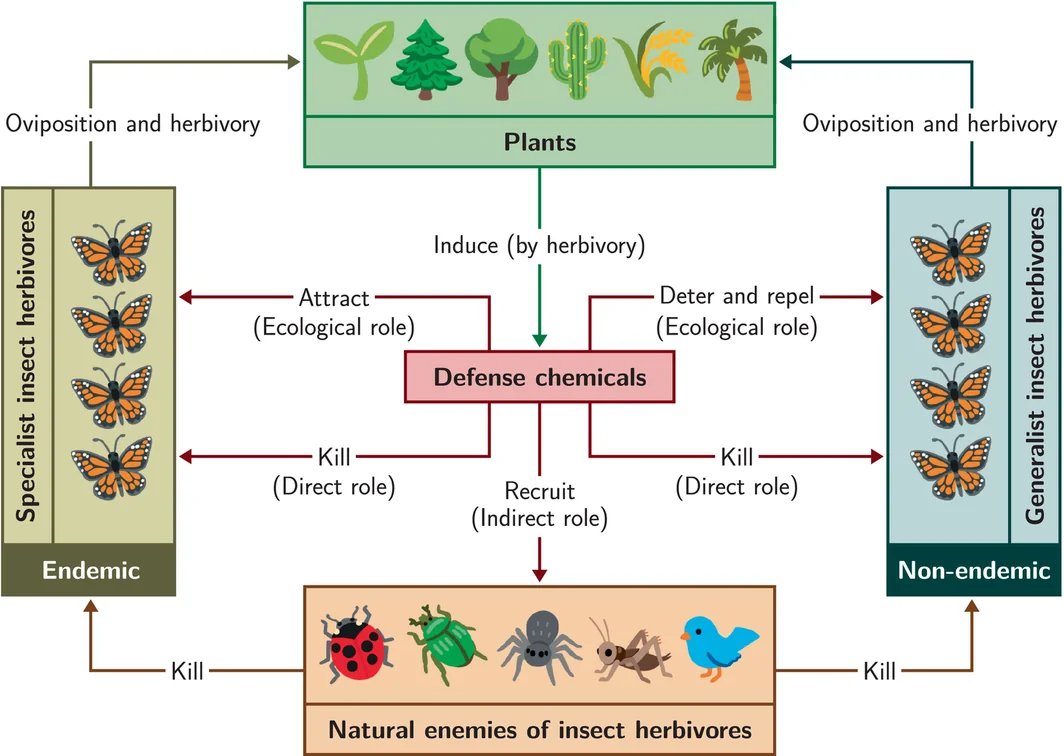
Multifaceted functions of plant defense compounds. Their ecological roles involve deterrence of generalists, attraction of specialists, and the recruitment of natural enemies to plants (indirect defense). The direct role of defense is to kill both specialist and generalist insects.

In a theoretical framework, we investigate how induced defense affects the specialist and generalist populations. To that end, we use deterministic models based on nonautonomous ordinary differential equations (ODEs). Models in mathematical biology describe biological events using simplified equations (Murray 2002; Kot 2001; Martcheva 2015; Arditi and Ginzburg 1989). In our model, events in chemical ecology, that is, the direct and ecological roles, including the indirect role of plant defense compounds, are represented by using ODEs. The multifaceted roles of chemical defense are described in the schematic diagram in Figure 1. The direct roles are the negative effects on insect herbivores, i.e., slow development, less food consumption, and high insect mortality (Jeschke et al. 2017, 2021; Rohr et al. 2011). The ecological roles, on the other hand, control the oviposition behavior of insects and their population (Chakraborty et al. 2023a; Van Der Meijden 1996; Lankau 2007). For example, defense chemicals attract specialists, but deter generalists (Chakraborty et al. 2023a; Van Der Meijden 1996; Lankau 2007; Poelman et al. 2010), although many specialists are killed at high concentrations (War et al. 2019). The indirect role of defenses is also part of their ecological functions. Specifically, plants attract or recruit natural enemies (predatory wasps, parasitoids, birds, etc.), which kill insect herbivores (Hopkins et al. 2009; Dicke and Baldwin 2010; Thaler 1999). For example, glucosinolate breakdown products attract the parasitoid wasp *Cotesia rubecula* in cruciferous crops (Van Poecke et al. 2001; Reddy et al. 2002). As described in Figure 1, the induction of plant defense subsequently initiates tritrophic interactions (Heil 2008; Price et al. 2011). These are complex relationships between plants, insects, and their natural enemies mediated by induced secondary metabolites (Ito and Sakai 2009; Liu et al. 2009; Fergola and Wang 2011; Harvey et al. 2007). Therefore, it would be intriguing to establish a direct correlation between induced defense, insects, and natural enemies and, thus, to understand how plants minimize biotic stress (herbivory by specialists and generalists).

In contrast to constitutive defense, induced defense is interactive because defense induction occurs after plants detect an attack or herbivory (Textor and Gershenzon 2009; Dicke and Baldwin 2010; Moreira et al. 2018; Dicke 1998; Sharma et al. 2022). This makes induced responses, which are triggered by herbivory, more specific and dynamic (Ali and Agrawal 2012; Agrawal 2000; Danner et al. 2018). The triggers are mathematically defined by initial conditions (Chakraborty et al. 2023a; Murray 2002; Kot 2001), that is, the initial population sizes of specialists and generalists at the beginning of herbivory. In addition, constitutive defense could be optimal (Chakraborty et al. 2023a; Van Der Meijden 1996) but unstable. After a small perturbation, which in our case is the beginning of herbivory, constitutive defense may no longer be sufficient (Textor and Gershenzon 2009; Dicke and Baldwin 2010; Moreira et al. 2018; Dicke 1998). Induced plant defense, on the other hand, could provide stable solutions. Moreover, specialists can tolerate or metabolize the increased levels of defenses more efficiently and may even use that as a cue for foraging. This may even increase the population density of specialists. This, in turn, may result in even higher levels of induction. This latter feedback is modeled here implicitly only by allowing the defense chemicals to reach high levels.

Our model is influenced by the models of the tripartite interactions among plants, insect herbivores and natural enemies, based on nonautonomous ODEs (Chakraborty et al. 2023a) and a graphical method (Van Der Meijden 1996) published earlier. Instead of time, the level of defense is the independent variable, like in our previous model (Chakraborty et al. 2023a). Many plants accumulate high levels of defense chemicals, which—even as a constitutive defense—can constitute more than 10% of the dry weight in some organ, for example, peppercorns (Divekar et al. 2022; Iqbal and Poór 2025; Rodríguez‐Romero et al. 2022; Morquecho‐Contreras et al. 2017). Naturally, induced defense elevates these levels even further (Textor and Gershenzon 2009; Dicke and Baldwin 2010; Moreira et al. 2018; Dicke 1998; Sharma et al. 2022). Therefore, we present a model that provides an understanding of the effects of strong chemical defense on specialist and generalist insects.

The study focuses on explicitly explaining the induced defense of plants against specialists, which are endemic, and generalists, which are non‐endemic. In particular, by building two independent ODEs, we theoretically investigate whether the induced defense response against specialist herbivores is similar to or different from that against generalist herbivores. This is a long‐standing topic of debate (Ali and Agrawal 2012; Agrawal 2000; Danner et al. 2018; Poelman et al. 2008; Reymond et al. 2004).

In addition, our goal is to investigate whether, by inducing defense compounds to a high level, a plant can limit the insect population to asymptotically stable equilibria. We expect to find qualitative differences between the stable equilibria of specialists (Devictor et al. 2010; Büchi and Vuilleumier 2014; Kneitel 2018) versus generalists (Chakraborty et al. 2024; Devictor et al. 2010; Büchi and Vuilleumier 2014; Kneitel 2018). Finding the stability of the nonautonomous model is necessary because it maintains the stationary states despite perturbations and changes in the initial conditions (Jaeger and Monk 2014; Van Emmerik et al. 2016; Sayevand et al. 2018), which explains the existing real‐world situation.

## 2 Method and Results

The model includes one independent variable D and three dependent variables $S\left(D\right),G\left(D\right)$ and $N\left(D\right)$ (see also the related model published earlier (Chakraborty et al. 2023a)). The notation is as follows: D is the level of defense induced by herbivory in a plant, $S\left(D\right)$ is the specialist population attracted to the plant, $G\left(D\right)$ is the remaining generalist population on the plant and $N\left(D\right)$ is the population of natural enemies recruited in the plant by defense level D. Note that there is a constitutive defense in addition.

To formulate the model, it is necessary to explicitly define the initial conditions, especially to represent the induced defense phenomenon (Textor and Gershenzon 2009; Dicke and Baldwin 2010; Dicke 1998; Ali and Agrawal 2012; Halitschke et al. 2001). While constitutive plant defense is always present, induced plant defense represents an augmentation beyond the constitutive levels, in response to abiotic or biotic stress (Kessler 2015; Takshak and Agrawal 2019). Constitutive defense has previously been shown to minimize the selection pressure by both insect groups (specialists and generalists) to a non‐zero, that is, positive value (population number) (Chakraborty et al. 2023a; Van Der Meijden 1996). In order that induced defense (which is studied in this paper) is triggered, some herbivores must be present at the outset (Textor and Gershenzon 2009; Ali and Agrawal 2012). Therefore, the initial conditions for S and G are $S\left(D=0\right)=S_{0}>0$ and $G\left(D=0\right)=G_{0}>0$. In contrast, the initial condition for the population of natural enemies is $N\left(D=0\right)=N_{0}=0$ because no natural enemies are recruited if the induced defense concentration is zero (D=0).

The model includes the following parameters. In case of specialists, let α be the constant rate (per unit defense) at which they are attracted, η be the constant death rate (per unit defense and per specialist) of their population due to the direct effect of toxins, and μ be their constant death rate (per specialist and per natural enemy) due to predation and parasitism. In case of generalists, let β be the constant rate (per unit defense and per generalist) of their deterrence, ϕ be the constant death rate per unit defense and per generalist of their population, and ν be their constant death rate per generalist and per natural enemy. It is assumed that natural enemies are attracted to a plant at a constant rate γ. Therefore, the population of recruited natural enemies is simply proportional to the level of induced defense D. Note that the rate constants do not refer to time (Chakraborty et al. 2023a; Van Der Meijden 1996) but to induced defense because the independent variable is D. The nonautonomous ODEs representing the change in the specialist and generalist populations are as follows:

(1a) $$S^{\prime }=\frac{\mathrm{dS}}{\mathrm{dD}}=\mathrm{\alpha D}-\mathrm{\eta SD}-\mathrm{\mu SN}$$

(1b) $$G^{\prime }=\frac{\mathrm{dG}}{\mathrm{dD}}=-\mathrm{\beta GD}-\mathrm{\phi GD}-\mathrm{\nu GN}$$

(1c) $$N^{\prime }=\frac{\mathrm{dN}}{\mathrm{dD}}=\gamma \Rightarrow N=\mathrm{\gamma D},\;\text{because}\;N_{0}=0$$

These differential equations can be used to mimic, in an approximative way, the change in the populations of specialist and generalist herbivores as a function of the defense level.

The bilinear functions in equations (1a) and (1b) represent the outcomes through interactions between two variables (Murray 2002; Kot 2001; Martcheva 2015). For example, ηSD is the death of specialists due to the interaction with defense; μSN is the death of specialists due to the predation or parasitism natural enemies. In case of sequestering specialists that use stored toxins of plants against natural enemies, μ should be low (Sporer et al. 2021). βGD refers to the generalists deterred by the interaction with defense; ϕGD and νSN describe the death of generalists due to the interaction with defense and natural enemies, respectively. The construction of such bilinear functions is based on Lotka‐Volterra (Lotka 1925; Volterra 1928; Abakuks 1982) and epidemiological (Martcheva 2015; Kermack and McKendrick 1927) models. We can solve the differential equations starting from D=0 up to some final value of D.

**FIGURE 2:**
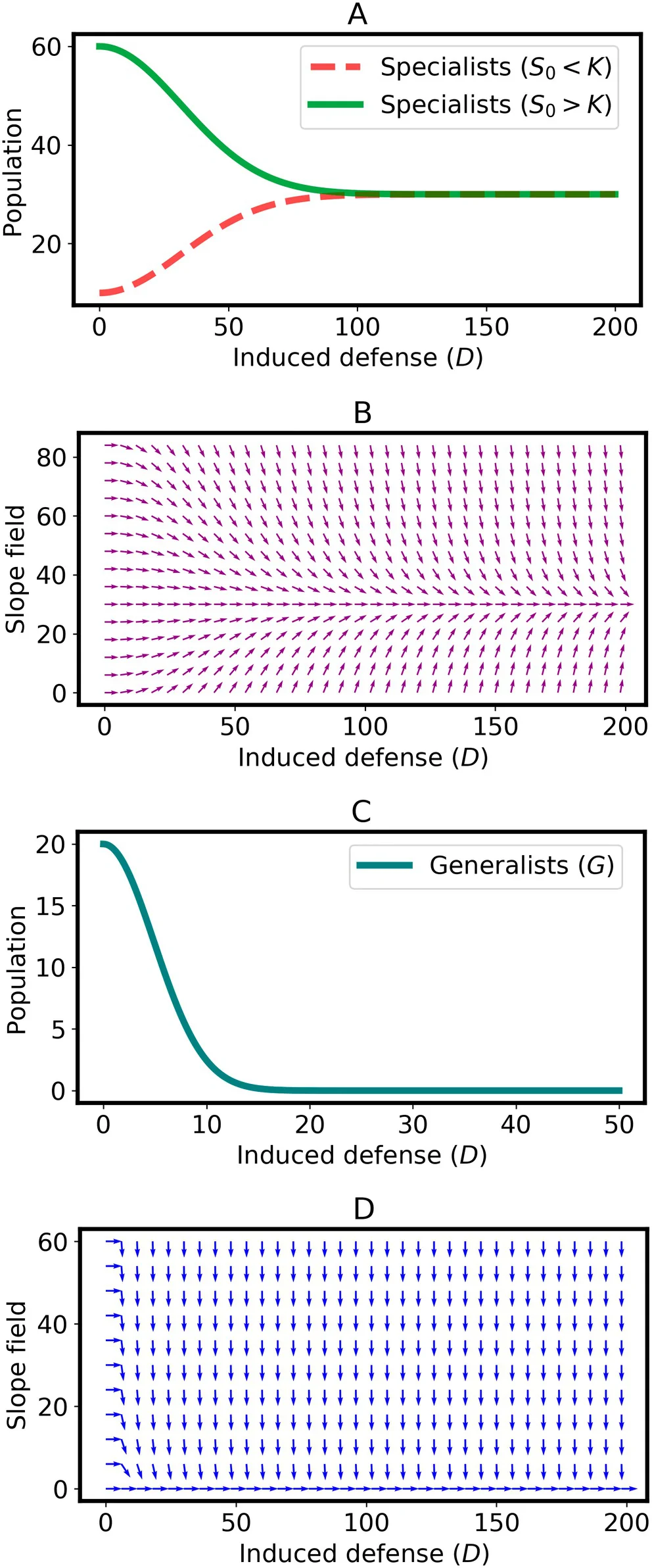
Population densities as functions of induced defense level D, as computed by using model 2. (A) Specialists with initial conditions 10 and 60. Parameters: α=0.03,θ=0.001,K=30. (B) Slope field plot of S indicating the derivative given in Equation (2a). Parameters are the same as in Figure 2A, initial conditions vary between 0 and 85. (C) Generalists. Parameter: δ=0.042, initial condition: $G_{0}=20$. (D) Slope field plot of G indicating the derivative given in Equation (2b). The parameter is the same as in Figure 2C, initial conditions vary between 0 and 60.

The dependent variables S and G are plotted with respect to the independent variable D in Figure 2 (A, C) for some definite parameter values. Replacing N=γD from equation (1c) to equations (1a) and (1b), the model 1 is simplified to the form:

(2a) $$S^{\prime }=\mathrm{\alpha D}\left(1-\frac{\mathrm{\theta S}}{\alpha }\right)=\mathrm{\alpha D}\left(1-\frac{S}{K}\right),\;\text{where}\;\theta =\eta +\mu \gamma \;\text{and}\;K=\frac{\alpha }{\theta }$$

(2b) $$G^{\prime }=-\mathrm{\delta GD},\;\text{where}\;\delta =\beta +\phi +\nu \gamma$$

The differential equations (2a) and (2b) can be solved analytically although they are nonautonomous, so that S and G can be written as functions of D:

(3a) $$S=K\;\left(1-e^{\frac{-\theta D^{2}}{2}}\right)+S_{0}e^{\frac{-\theta D^{2}}{2}}$$

(3b) $$G=G_{0}\;e^{-\frac{\delta D^{2}}{2}}$$

The calculation for the solutions (3a) and (3b) is provided in Appendix A. Although we want to analyze the behavior for finite D only, it is of interest to see to which values S and G tend for very large D. The solution of Equation (3a) converges to the equilibrium S=K and Equation (3b) converges to the equilibrium G=0 as D→∞ (understood as a mathematical idealization of the case of very high defense), as proved in Appendix A. Therefore, the stability of the equilibria is obtained as D becomes large.

The significance of the model 2 is as follows:

1. Equations (2a) and (2b) are independent of each other because the interaction between specialists and generalists is not included in our model. Equation (2a) has the asymptotically stable equilibrium S=K>0 and an extremum at D=0, as can be seen from the analytical solution given in Equation (3a) (see also Figure 2A,B). K determines an effective (defense‐induced) carrying capacity of the attracted specialist population, because it represents their stable population size. This can be interpreted in that specialist insects should be endemic herbivores, which is the real‐world situation. Similarly, Equation (2b) has the equilibrium G=0 and an extremum at D=0 (Equation (3b)).
2. The derivative $S^{\prime }$ can be negative or positive until the equilibrium point K is reached, as shown in Figure 2A,B. It is reached like a plateau quite fast for finite D values, due to the quadratic term in the exponent in Equation (3a). If $S_{0}>K$ then $S^{\prime }<0$, that is, strictly monotonic decreasing S (Figure 2A,B), while if $S_{0}<K$, then $S^{\prime }>0$, that is, strictly monotonic increasing S (Figure 2A,B). The stability of that equilibrium point follows from the analytical solution in Equation (3a) and is shown in the slope field plot (Figure 2B). The case $S_{0}>K$ where the specialist population decreases to the effective carrying capacity, K (Figure 2A,B) is realistic because plants induce toxins to minimize herbivory. In the case $S_{0}<K$, in contrast, the population (possibly of a different specialist) increases to finally reach K. This could be a hypothetical situation where the initial population density of specialists is low and/or the attraction rate of specialists, α, is much higher than their death rate, θ, by direct and indirect functions of defense compounds (Figure 2A,B). It could occur if a plant produces a defense to reduce generalists and—as an adverse effect—attracts specialists by that.
3. The derivative $G^{\prime }$ is negative (i.e., strictly monotonic decreasing G) until the equilibrium point G=0 is reached, which is also the minimum point (Figure 2C). The stability of that equilibrium point follows from the analytical solution in Equation (3b) and is shown in the slope field plot (Figure 2D). The maximum population of generalists is given by the initial value $G_{0}$ at D=0. The equilibrium G=0 is asymptotically stable and is reached quite fast as D increases, due to the quadratic term in the exponent in Equation (3b). Thus, it can be concluded that generalist insects are non‐endemic herbivores of a plant or only occur in low numbers due to the induced defense. Note that the solution for G is, in the positive orthant, similar to the Gaussian distribution. However, it does not describe a statistical distribution here.

## 3 Discussion

In a previous paper (Chakraborty et al. 2023a), we used mathematical modeling to study constitutive plant defense. Here, we have focussed on induced defense. As mentioned in the Introduction, induced defense chemicals can reach high concentrations. Several experimental studies previously reported induced defense in plants (Durrant et al. 2017; Rosa‐Diaz et al. 2024; Kundu et al. 2025), where the concentrations of secondary metabolites are increased above their constitutive levels (Textor and Gershenzon 2009; Ali and Agrawal 2012). Therefore, it is of interest to analyze what happens when the defense, D, is increased more and more, up to levels that the plant can still afford. An interesting result of our simulations is that the population densities of specialist and generalists reach positive and zero plateau levels, respectively, which can be interpreted as equilibria. To check their stability, it is meaningful to consider the hypothetical case that D increaes even more. That the equilibria found in this study are stable is of interest because this could contribute to balanced states in an ecosystem, which cannot be affected by minor disturbances (Van Emmerik et al. 2016; Sayevand et al. 2018; Böttcher et al. 2023; Zhou 2013). Therefore, from the stable equilibria S=K and G=0, the main results of this study can be interpreted as follows.

- Plants limit the specialist insect population to a certain effective carrying capacity (K>0) by inducing defense compounds, because S=K is a (globally) asymptotically stable equilibrium. Therefore, specialists are endemic in the wild in spite of herbivory‐induced plant defense. The real benefit of inducing toxins in a plant is to reduce the number of specialists from a high initial value to a low carrying capacity (K) value, Figure 2A. However, if the initial population density of specialists is low, also the opposite scenario is possible. While this would backfire to the plants, it has at least the advantage of repelling generalists.
- If K is low, then the number of specialist herbivores tends to a low value, which could occur if α≪θ, that is, the attraction rate of specialists is much lower than the death rate of specialists by direct and indirect functions of defense compounds.
- Plants reduce the generalist insect population to a very low value of even zero by inducing defense compounds, because G=0 is an asymptotically stable (globally) equilibrium. The exact value depends on the final value of D. Due to the sharp decrease of the bell‐shaped function $G\left(D\right)$, which is due to the quadratic term in the exponent, G levels off at zero for moderate defense levels. So generalists are practically non‐endemic in the wild in the sense that they cannot stay in a habitat for a longer time because plants induce defense compounds (see also Chakraborty et al. 2024). It is worth mentioning that the asymptotic stability of G=0 does not guarantee that generalists become extinct because they can leave their host plant after initiating an attack (note the deterrence and other terms in Equation (1b)).
- That S and G converge to the equilibria S=K and G=0 when D becomes large may elucidate the reason why plants in the wild induce defense chemicals to a relatively high level, in spite of the fact that these are secondary metabolites (Ali and Agrawal 2012; Poelman et al. 2008; Reymond et al. 2004). The final level also depends on the toxicity of the particular chemical. The higher its toxicity is, the lower could be the necessary concentration.

For the sake of simplicity, we have used differential equations containing linear and bilinear terms. In particular, the changes in the population densities depend linearly on the defense level, D. Utilizing nonlinear functions would not necessarily alter the qualitative result that the population densities reach plateau values, which could similarly be interpreted as saturation. Moreover, it is crucial to distinguish between the immediate effect at any time point (e.g., on growth rate) and the cumulative effect. Empirical data regarding the former effect are frequently captured by linear functions (Agrawal and Kurashige 2003). This is not contradictory to the observation that the cumulative effect curves (Figure 2A,C) resemble Hill inhibition curves as often resulting from dose–response relations for toxins (Gadagkar and Call 2014); indeed, it is entirely consistent with it. Thus, our model offers an alternative explanation for this kinetics, beyond the traditional framework of cooperativity.

Debate persists about whether plants exhibit differential defense induction against specialists versus generalists (Ali and Agrawal 2012). For example, some studies reported distinct induced resistance against these two types in Brassicaceae plants (Ali and Agrawal 2012; Agrawal 2000; Danner et al. 2018), while other studies reported that both insect groups elicited a similar induced response (Ali and Agrawal 2012; Poelman et al. 2008; Reymond et al. 2004). In this study, we use the simplification that plants would use the same type of chemical defense—which may be a mixture of several substances—against specialists and generalists. We theoretically explain that a substantial defense is induced regardless of the attacking insect group, that is, a similar response against both insect groups as suggested by some experimental studies published before (Ali and Agrawal 2012; Poelman et al. 2008; Reymond et al. 2004). However, even with the same induced defense by plants, the outcomes for specialists and generalists are completely different.

Although stability is usually analyzed in a dynamical system (Murray 2002; Kot 2001; Martcheva 2015), we have not formulated a dynamical system because the independent variable (in ODEs) is induced defense rather than time (Chakraborty et al. 2023a; Van Der Meijden 1996; Bellman 2008). There is a fitness cost associated with the production of secondary metabolites (Van Der Meijden 1996; Ito and Sakai 2009), especially from the perspective of game theory (Denison et al. 2003; Dwivedi et al. 2025, 2023). However, the costs are invested by the plants, for which we did not write a differential equation. We only model the population densities of the (specialist and generalist) insects. The costs affect to what maximum level the plant can increase the defense level, D. In Figure 2A,C, it can then be seen which population densities are reached for a given finite D (partly determined by the costs).

In many cases, the level of induced defense begins to decline once herbivory is sufficiently reduced (Karban and Myers 1989; Züst and Agrawal 2017)‐ particularly as plateau values are reached—allowing any overshoot of D to be reversed. If the variables S and G are unique functions of the defense level and independent of the history of events, our current model can already account for this. D would simply “turn” and decrease, with herbivore population densities retracing their previous states. In contrast, if the dynamics are history‐dependent (hysteresis), a more complex model involving a parametric curve would be required. Such an extension represents a compelling task for future refinements of the model.

In mathematical biology or ecology, autonomous dynamical systems are commonly applied. In contrast, the occurrence of nonautonomous ODEs is less common, but they could be more inclusive. One can analyze both the equilibrium and extremum points (defined by time or independent variable) in a qualitative nonautonomous system (Chakraborty et al. 2023a; Van Der Meijden 1996; Cheban 2020), while a qualitative autonomous dynamical system does not explicitly take into account the role of time (Murray 2002; Kot 2001; Martcheva 2015). In addition, the qualitative theory for the asymptotic stability of nonautonomous systems could be more complex (Aeyels 1995; Barreira and Valls 2008). In this study; however, an analytical nonautonomous model (1) is built, where stability analysis is quite straightforward, explained by directly plotting the slope fields in Figure 2B,D. Advancements in the model (1) could be made in the future, which may provide a qualitative system that requires an extension of stability analysis (Aeyels 1995; Barreira and Valls 2008).

A major point of this paper is to establish that chemical ecology is an integral part of community ecology (Krebs et al. 2025; Søndergaard et al. 2025) because the induced defense caused by biotic (or abiotic) stress influences the interaction of different insect groups with a specific host plant (Chakraborty et al. 2023a; Van Der Meijden 1996; Lankau 2007). In particular, it is clear from our results that specialists have primary hosts because they are endemic (Figure 2A). Thus, they coexist with their host plants. However, generalists only have temporary hosts because they are non‐endemic (Figure 2C). Therefore, they feed on different host plant families for their existence. In this way, the overall functioning of an ecosystem is mediated by secondary metabolites. That could also explain why secondary metabolites independently provide novel community structures (Krebs et al. 2025; Søndergaard et al. 2025), regardless of vital dynamics, that is, without considering time‐dependent rates of birth and death (Demain and Fang 2000; Wink 2018). However, vital dynamics could be important to quantify variability in the existing populations (Murray 2002; Kot 2001; Martcheva 2015). That could be described in the future by building models based on partial differential equations (PDEs) (Jost 2013; Borthwick 2017) with time as an independent variable in addition to the induced defense or by relatively simpler models based on ODEs, where the induced defense could be transformed into a function of time. Moreover, it would be intriguing to expand the model (2) from an individual plant to the general population of plants to obtain a holistic understanding of ecological niche and community dynamics (Krebs et al. 2025; Søndergaard et al. 2025; Pocheville 2015).

## Author Contributions

**Suman Chakraborty:** conceptualization (lead), data curation (lead), formal analysis (lead), investigation (lead), methodology (lead), software (lead), validation (equal), visualization (lead), writing – original draft (lead), writing – review and editing (equal). **Stefan Schuster:** formal analysis (supporting), funding acquisition (lead), investigation (supporting), methodology (supporting), project administration (lead), supervision (lead), validation (equal), writing – review and editing (equal).

## Funding

This work was supported by Deutsche Forschungsgemeinschaft, 239748522.

## Conflicts of Interest

The authors declare no conflicts of interest regarding this manuscript.

## Data Availability

All data is available in the manuscript.

## Appendix A

Solution of equation (2a)

$$\begin{array}{c} \frac{\mathrm{dS}}{\mathrm{dD}}=\mathrm{\alpha D}\left(1-\frac{S}{K}\right)\\ \;\Rightarrow \int \frac{\mathrm{dS}}{1-\frac{S}{K}}=\int \mathrm{\alpha D}\;\mathrm{dD}\\ \;\Rightarrow \int \frac{\mathrm{KdP}}{P}=-\int \mathrm{\alpha D}\;\mathrm{dD},\;\text{where}\;P=1-\frac{S}{K}\Rightarrow \mathrm{dS}=-\mathrm{KdP}\\ \;\Rightarrow K\ln P=-\frac{\alpha D^{2}}{2}+C,\;\text{where}\;C\;\text{is an arbitrary constant}\\ \;\Rightarrow K\ln\left(1-\frac{S}{K}\right)=-\frac{\alpha D^{2}}{2}+K\ln\left(1-\frac{S_{0}}{K}\right),\;\text{where}\;C=K\ln\left(1-\frac{S_{0}}{K}\right)\\ \;\Rightarrow \frac{1-\frac{S}{K}}{1-\frac{S_{0}}{K}}=e^{-\frac{\theta D^{2}}{2}}\\ \;\Rightarrow \frac{K-S}{K-S_{0}}=e^{-\frac{\theta D^{2}}{2}}\\ \;\Rightarrow S=K-\left(K-S_{0}\right)e^{-\frac{\theta D^{2}}{2}}\\ \;\Rightarrow S=K\left(1-e^{-\frac{\theta D^{2}}{2}}\right)+S_{0}e^{-\frac{\theta D^{2}}{2}}, \end{array}$$

which is the solution given in equation (3a).

Solution of equation (2b)

$$\begin{array}{c} \frac{\mathrm{dG}}{\mathrm{dD}}=-\mathrm{\delta GD}\\ \;\Rightarrow \int \frac{\mathrm{dG}}{G}=-\int \mathrm{\delta D}\;\mathrm{dD}\\ \;\Rightarrow \ln G=-\frac{\delta D^{2}}{2}+L,\;\text{where}\;L\;\text{is an arbitrary constant}\\ \;\Rightarrow \ln G=-\frac{\delta D^{2}}{2}+\ln G_{0},\;\text{where}\;L=\ln G_{0}\\ \;\Rightarrow G=G_{0}\;e^{-\frac{\delta D^{2}}{2}}, \end{array}$$

which is the solution given in equation (3b).

Convergence of S and G

$$\begin{array}{c} S\;\text{converges to}\;K,\text{because}\lim_{D\to \infty }S=\lim_{D\to \infty }\left(K\;\left(1-e^{\frac{-\theta D^{2}}{2}}\right)+S_{0}e^{\frac{-\theta D^{2}}{2}}\right)=K. \end{array}$$

$$\begin{array}{c} G\;\text{converges to}\;0,\text{because}\lim_{D\to \infty }G=\lim_{D\to \infty }G_{0}\;e^{-\frac{\delta D^{2}}{2}}=0. \end{array}$$
